# Role of Mitochondrial Dynamics in Heart Diseases

**DOI:** 10.3390/genes14101876

**Published:** 2023-09-26

**Authors:** Takeshi Tokuyama, Shigeru Yanagi

**Affiliations:** 1 Division of Regenerative Medicine, Center for Molecular Medicine, Jichi Medical University, Shimotsuke 329-0498, Tochigi, Japan; 2 Laboratory of Molecular Biochemistry, Department of Life Science, Faculty of Science, Gakushuin University, Mejiro, Tokyo 171-0031, Japan; shigeru.yanagi@gakushuin.ac.jp

**Keywords:** mitochondrial dynamics, fission and fusion, heart failure, cardiovascular diseases, Drp1, Mfn1, Mfn2, Opa1

## Abstract

Mitochondrial dynamics, including fission and fusion processes, are essential for heart health. Mitochondria, the powerhouses of cells, maintain their integrity through continuous cycles of biogenesis, fission, fusion, and degradation. Mitochondria are relatively immobile in the adult heart, but their morphological changes due to mitochondrial morphology factors are critical for cellular functions such as energy production, organelle integrity, and stress response. Mitochondrial fusion proteins, particularly Mfn1/2 and Opa1, play multiple roles beyond their pro-fusion effects, such as endoplasmic reticulum tethering, mitophagy, cristae remodeling, and apoptosis regulation. On the other hand, the fission process, regulated by proteins such as Drp1, Fis1, Mff and MiD49/51, is essential to eliminate damaged mitochondria via mitophagy and to ensure proper cell division. In the cardiac system, dysregulation of mitochondrial dynamics has been shown to cause cardiac hypertrophy, heart failure, ischemia/reperfusion injury, and various cardiac diseases, including metabolic and inherited cardiomyopathies. In addition, mitochondrial dysfunction associated with oxidative stress has been implicated in atherosclerosis, hypertension and pulmonary hypertension. Therefore, understanding and regulating mitochondrial dynamics is a promising therapeutic tool in cardiac diseases. This review summarizes the role of mitochondrial morphology in heart diseases for each mitochondrial morphology regulatory gene, and their potential as therapeutic targets to heart diseases.

## 1. Introduction

Mitochondria are essential organelles for adenosine triphosphate (ATP) production and are closely linked to cellular health and energy demand. On the other hand, mitochondria release reactive oxygen species (ROS) that cause oxidative damage. These mitochondrial functions are regulated by two distinct systems: fission and fusion. Fission, facilitated by proteins such as dynamin-related protein 1 (Drp1), fission 1 (Fis1), mitochondrial fission factor (Mff), and mitochondrial division (MiD) 49 and 51, leads to mitochondrial fragmentation, which is essential for cell division and removal of damaged mitochondria. Fusion, orchestrated by mitofusin 1 (Mfn1), mitofusin 2 (Mfn2), and optic atrophy 1 (Opa1), results in interconnected networks that help repair mitochondrial DNA and distribute energy. While the roles of fission proteins are primarily associated with morphological changes, fusion proteins show diverse roles influencing mitophagy, apoptosis, and energy production. Consequently, mitochondrial morphology is strictly regulated by several key genes. Unlike other cells, the adult heart contains relatively static mitochondria that are segregated into three functional subpopulations. Despite this fixed arrangement, emerging evidence suggests that mitochondrial morphological factors play an important role in cardiac health and pathology.

Mitochondrial dysfunction has been implicated in a spectrum of cardiac diseases. Defective fission has been associated with severe cardiac abnormalities, highlighting the importance of these dynamics in cardiac pathologies, including cardiomyopathies and ischemic conditions. A profound understanding of mitochondrial dynamics opens a new avenue for therapeutic approaches to heart disease. The balance between fission and fusion processes offers a promising strategy to preserve cardiac health, especially under stress.

In this review, we summarize the relationship between mitochondrial morphology regulatory genes and heart diseases. We discuss the molecular biology underlying mitochondrial dynamics, clarify how defects in mitochondrial dynamics are associated with heart damage during stress, and detail the importance of considering mitochondrial dynamics as a new therapeutic target for mitochondrial morphology regulatory genes.

## 2. Mitochondrial Morphology

Mitochondrial dynamics involves a continuous process of mitochondrial fission and fusion. Mitochondrial dynamics regulate mitochondrial turnover and the intracellular environment [1]. Here, we summarize the basic knowledge of the regulation of mitochondrial morphology.

### 2.1. Mitochondrial Fission

Mitochondrial fission depends on the activation of Drp1 in the cytoplasm, which causes Drp1 to translocate into the mitochondria and bind to receptors on the mitochondrial outer membrane (OMM). Mitochondrial fission produces daughter mitochondria with different membrane potentials (Δψm). This function supports the selective degradation of damaged mitochondria by autophagy and maintains cellular homeostasis [2,3]. During mitosis, fission ensures the distribution of damaged mitochondria to daughter cells [4]. Drp1 mainly regulates mitochondrial fragmentation in mammals and interacts with several organelles [5]. Dynamin 2 is involved in mitochondrial fragmentation [6], but its deletion does not stop mitochondrial fragmentation [7,8]. Thus, Drp1 is essential for membrane contraction and separation [9]. Once translocated into the mitochondria, Drp1 aggregates and surrounds the mitochondria, causing mitochondrial fission [10,11,12,13]. The endoplasmic reticulum (ER) influences and constricts the sites that will align Drp1 rings to divide mitochondria [10,14,15]. As the ER membrane wraps around mitochondria, actin associates primarily with Formin 2 on the ER surface. Formin 2 cooperates with Spire1C to promote actin assembly at the mitochondrial surface [16] and also communicates with myosin II [17,18,19]. When fission is triggered, actin groups on the OMM turn on Drp1, which is translocated to the mitochondria. Fission requires actin to form chains and apply tension to the membrane [20,21].

Drp1, a key fission protein, is regulated by multiple post-translational modifications, including phosphorylation [22,23,24], ubiquitination [25], SUMOylation [26], O-GlcNAcylation [27], and nitrosylation [14,28]. Phosphorylation, particularly at S637 and S616, plays an important role. Protein kinase A phosphorylates S637 in response to cAMP and inhibits fission by blocking its GTPase activity [23,24]. The phosphorylation of Drp1 at the Ser 616 (S616) site, mediated by cyclin-dependent kinase (CDK) 1/Cyclin B or CDK5, facilitates mitochondrial fission during mitosis [29,30]. Drp1 is regulated by transcription and protein degradation. Small RNA molecules such as miR can alter the *Drp1* gene expression. During apoptosis, RNA known as miR-30 is reduced, and *Drp1* expression is increased [31]. In myocardial infarction, miR-499 regulates calcineurin and acts to protect the heart [32]. Drp1 degradation affects mitochondrial fragmentation. When Parkin is deleted, Drp1 increases, and fission is accelerated [33]. Mitochondrial ubiquitin ligase (MITOL/MARCH5) interacts with Drp1 to affect fragmentation [34,35]. MITOL/MARCH5 deficiency in the heart causes heart failure with increased mitochondrial fragmentation in mice [36].

#### Receptors Required for Drp1 Recruitment

**Figure 1:**
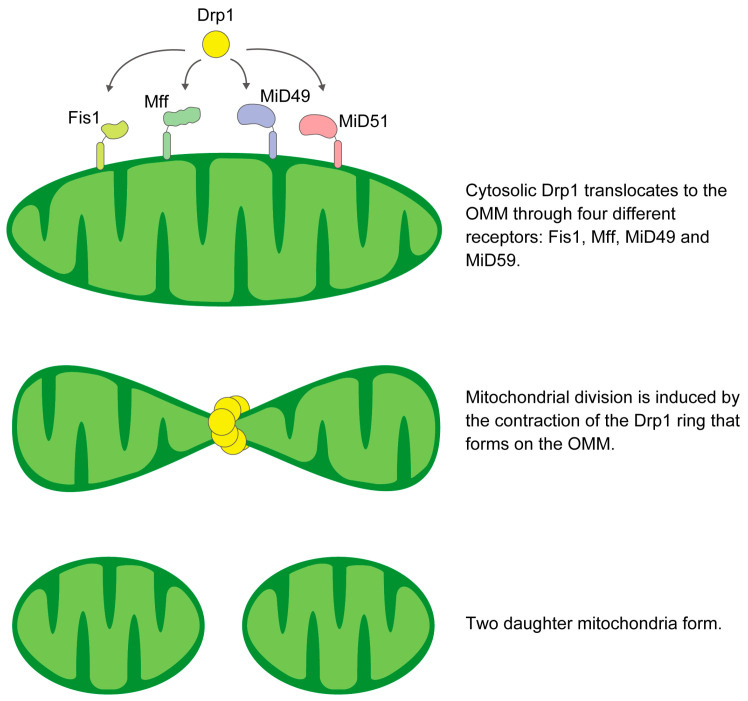
Schematic illustration of the mechanism of mitochondrial fission.

In mammals, Drp1 recruitment to the OMM depends on key adaptors: Fis1, Mff, and MiD 49 and 51 [37,38,39]. These adaptors are essential for mitochondrial fragmentation by Drp1. Fis1 is a protein anchored at its C-terminus to the OMM, exposing a 15 kDa soluble domain to the cytosol [40,41,42] (Figure 1). Fis1 was first identified as a Drp1 adaptor in mammalian cells. Although Fis1 recruits Drp1 in specific cells or situations, its overexpression consistently induces mitochondrial fragmentation in mammalian cells [38,42,43,44,45,46,47]. This may be due to its ability to bind and repress fusion proteins or to mitochondrial dysfunction and Ca2+ overload [48,49]. It has been proposed that Fis1 not only acts as a Drp1 receptor but mainly promotes lysosomal recruitment of Drp1 for peripheral fission and mitophagy [3,45,50]. This is consistent with the role of Fis1 in regulating mitochondria-lysosome contact, as exemplified by its association with Tbc1d15 [51,52].

Mff, which is anchored to the OMM, has a putative Drp1 binding domain at its N-terminus [37,38]. Inhibition of Mff expression attenuates both Drp1 recruitment and subsequent fission events [37,38]. Mff mediates the recruitment of Drp1 primarily in the mitochondrial midzone [3]. Under conditions of mitochondrial dysfunction and elevated AMP levels, protein kinase AMP-activated (AMPK) phosphorylates Mff and enhances its pro-fission activity [53].

MiD49 and MiD51 are mitochondrial adaptors of Drp1 that promote fission [54]. However, high levels of these adaptors can trap Drp1 and inhibit mitochondrial fragmentation [55,56]. The cryo-EM structure of the Drp1-MiD49 complex shows that after co-assembly, detachment of the MiD receptor occurs due to guanosine triphosphate (GTP) hydrolysis and exchange, resulting in a shortened filament and a contracted Drp1 ring. Therefore, an abundance of these receptors may not enhance fission, but their effect could be modulated by the precise timing of assembly and disassociation [57].

### 2.2. Mitochondrial Fusion

**Figure 2:**
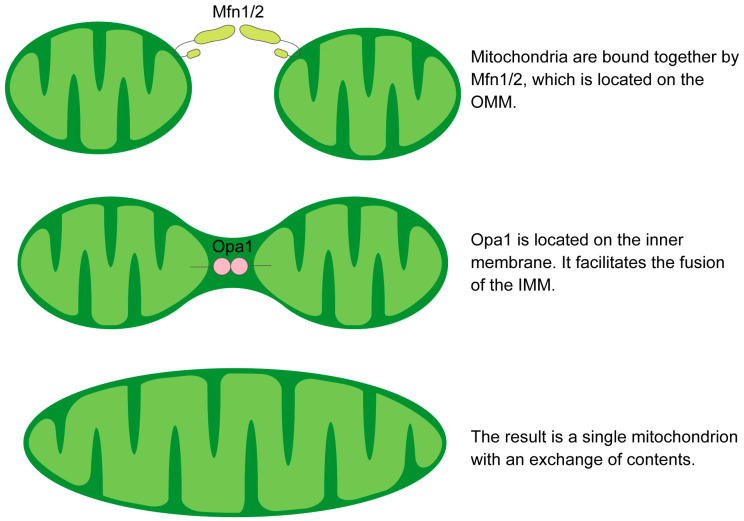
Schematic illustration of the mechanism of mitochondrial fusion.

The fusion process of the OMM is orchestrated by a GTPase-driven mechanism that is predominantly mediated by the homo- or hetero-oligomeric interactions of the mitofusins [58,59,60]. The subsequent inner mitochondrial membrane (IMM) fusion is mediated by Opa1, allowing the exchange of matrix components such as mtDNA, lipids and proteins between fused mitochondria [61,62]. Mfns are prevalent at contact points between neighboring mitochondria, facilitating tethering, docking, and extension of OMM contacts through either homotypic or more effective heterotypic complexes [63] (Figure 2). The presence of these heterotypic complexes means that overexpression of one protein can compensate for the loss of the other [64,65]. An increase in either protein leads to extensive mitochondrial fusion, resulting in elongated mitochondria. Among the Mfns, Mfn1 exhibits greater efficiency in docking and tethering, which is attributed to its superior GTPase activity compared to Mfn2 [60,63]. Mfn2 is essential for tethering mitochondria to the ER and other ER-derived organelles, crucial for Ca2+ and phosphatidylserine (PS) transfer [66,67]. This transfer supports phospholipid synthesis and influences membrane fission and fusion dynamics, particularly Drp1-mediated processes [8].

Mfns are regulated at multiple levels to control fusion. The peroxisome proliferator-activated receptor γ coactivator 1-α (Pgc-1α), estrogen-related receptor-α (Err-α), and Pgc-1β increase *Mfn2* expression [68], whereas the transcription factor Mef2 degradation during neuronal excitotoxicity decreases *Mfn2* expression [69]. Several miRNAs, including MiR-214 and MiR-106b, affect mitochondrial morphology by targeting *Mfns* [70,71]. Mitofusins undergo post-translational modifications such as oxidation, leading to Mfn oligomerization [72]. MITOL/MARCH5 ubiquitinates Mfn1 during stress [73], and histone deacetylase 6 (HDAC6) supports Mfn1 function during glucose shortage [74]. Various phosphorylation events can either support or inhibit the role of Mfns in fusion, such as ERK-mediated modification of Mfn1 [75,76]. These regulatory processes underscore the critical nature of mitochondrial dynamics in cellular function.

Opa1 is the only dynamin-like protein in the IMM and is essential for mitochondrial elongation [77]. Opa1 has eight splice variants. It’s processed at three sites by the metalloproteases YME1, like 1 ATPase (YME1L1) and M-AAA protease 1 (OMA1). Further cleavage by PARL produces a soluble intermembrane space (IMS) fraction that, in combination with IMM-anchored l-Opa1, maintains tight cristae junctions [78]. The human *OPA1* gene contains more than 30 exons and eight mRNAs that differ in biological function, such as maintaining mitochondrial fusion [79,80]. Mammalian Opa1 is mainly regulated by protease cleavage by Oma1 and Yme1l. Excess s-Opa1 inhibits fusion, whereas l-Opa1 can maintain fusion under stress [81,82]. The role of s-Opa1 in fission remains controversial, but it is known that both need to work simultaneously for effective fusion [83].

## 3. Dynamics of Mitochondria in Cardiomyocytes

**Figure 3:**
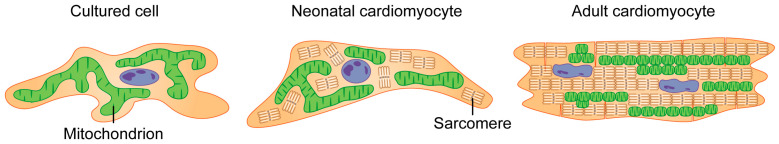
Mitochondrial morphology variation among cells. In cultured cells such as HEK293, neonatal cardiomyocytes and adult cardiomyocytes, mitochondrial morphology is different in the cells. Mitochondrial morphology in neonatal cardiomyocytes is similar to cultured cells. In contrast, adult cardiomyocytes are characterized by smaller, segmented mitochondria.

In cultured cells and neonatal cardiomyocytes, mitochondria are dynamic organelles that spread throughout the cytoplasm in a network-like pattern. In adult cardiomyocytes, however, mitochondria have a different arrangement, being more compact and tightly interconnected (Figure 3). Thus, the general hypothesis of mitochondrial morphology that mitochondrial fragmentation is detrimental and mitochondrial fusion is beneficial should be carefully applied to adult cardiomyocytes under physiological conditions [84]. Mitochondria in adult cardiomyocytes can be divided into three main types based on their location and function. Most mitochondria, called interfibrillar mitochondria (IFM), are located near the myofibrils and are essential for calcium signaling and as a source of energy for contraction. A smaller group, the sarcoplasmic submembrane mitochondria (SSM), are located near the sub-coronary artery membrane and are essential for ion channel energy and cell signaling. Another group, the perinuclear mitochondria (PNM), are located near the nucleus and support the energy required for transcription [85,86]. Recent studies have shown that despite their unique structure, mitochondria can change shape in adult cardiomyocytes [87,88,89,90,91,92]. This was observed by briefly tracking the movement of various fluorescent markers using tools such as electron microscopy and confocal microscopy [93,94,95,96,97]. These markers range from light-activated proteins to pH-sensitive probes [98,99,100,101,102,103,104,105,106]. Although many observational techniques are revealing mitochondrial dynamics in cardiomyocytes, these results differ from the basic mitochondrial dynamics obtained from cultured cells, and more studies in the heart and adult cardiomyocytes are needed to study mitochondrial dynamics in cardiomyocytes.

## 4. Animal Models of Mitochondrial Dynamics

Mitochondrial dynamics during heart development and in the resting state have been studied using whole-body or heart-specific knockout models targeting mitochondrial fission and fusion (Table 1). Here, we show the phenotypes of the heart driven by each genetic engineering of mitochondrial morphology factors.

### 4.1. Drp1

Deletion of the *Drp1* gene results in embryonic death by day E12.5 [107]. In the adult heart, loss of Drp1 disrupts mitophagy and causes cardiomyopathy [108]. This underscores the essential function of Drp1 in ensuring a robust mitochondrial network. In mice lacking Drp1, specifically in cardiomyocytes, there is a marked reduction in lifespan accompanied by mitochondrial respiratory dysfunction and accumulation of ubiquitinated proteins [109,110]. Furthermore, postnatal ablation of myocardial Drp1 leads to increased mortality [111]. In adult cardiomyocytes, deletion of Drp1 leads to upregulation of Parkin, resulting in increased mitophagy. This overactivity contributes to the onset of lethal cardiomyopathy. Simultaneous cardiac-specific deletion of both Drp1 and Parkin ameliorates cardiac remodeling and improves survival, underscoring the critical role of Parkin in regulating baseline mitophagic quality control [112]. In adult mice subjected to inducible cardiomyocyte-specific Drp1 deletion, dilated cardiomyopathy is developed under unstressed conditions, leading to mortality within 13 days. Cellular analyses reveal the presence of elongated and damaged mitochondria, reduced autophagy and increased cell death [108,111]. Overexpression of Drp1 results in mitochondrial fragmentation, while other proteins involved in mitochondrial dynamics remain unaffected. These fragmented mitochondria retain a typical cristae structure and maintain regular mitochondrial respiration. Thus, persistent Drp1-induced hyper-fragmentation does not inherently damage cardiomyocyte mitochondria or the mammalian heart [113]. Taken together, these findings suggest that a state of mitochondrial hyperactivity closely associated with Drp1 abnormalities is a strong predisposing factor for the development of hypertrophic cardiomyopathy.

### 4.2. Mfn1 and Mfn2

In mice, combined cardiac deletion of Mfn1 and Mfn2 results in spherical and functionally normal mitochondrial function, eventually leading to cardiac dysfunction by postnatal day 7 and death within 16 days of birth [114]. Combined ablation of tamoxifen-induced Mfn1/2 in adult cardiomyocytes causes mitochondrial fragmentation and dysfunction, leading to eccentric hypertrophy and lethal dilated cardiomyopathy [111,115,116]. In mice with cardiomyocyte-specific deletion of Mfn1, cardiac function and mitochondrial respiration remain intact, though spherical mitochondria are observed [117,118]. In contrast, mice with cardiomyocyte-specific Mfn2 ablation develop dilated cardiomyopathy [118], preceded by an initial phase of mild hypertrophy [119]. Cardiomyocyte-specific knockout models of Mfn1 and Mfn2 show different phenotypic outcomes, which can be explained by several factors. Primarily, Mfn2 functions as a Parkin receptor in addition to its overlapping roles with Mfn1. In addition, only Mfn2 is involved in tethering the endoplasmic reticulum (ER) to mitochondria, a critical interaction for mitochondrial energy metabolism and calcium regulation [66]. Therefore, in addition to mitophagy perturbations, Mfn2 knockout cardiomyocytes exhibit disruptions in calcium-related pathways due to decreased mitochondrial-sarcoplasmic reticulum (SR) tethering and impaired calcium uptake [67,120]. 

In Drosophila, knockdown of the cardiac genes *Marf* (human *Mfn*) and *Opa1* results in cardiomyopathy and reduces contractility. This effect is rescued by overexpression of human mitofusins [121]. In contrast to cardiomyocytes with fusion defects due to Mfn1/Mfn2 knockout or fission defects due to Drp1 knockout, the Mfn1/Mfn2/Drp1 triple knockout cardiomyocytes exhibit prolonged survival and overt cardiac hypertrophy. The simultaneous disruption of mitochondrial fission and fusion leads to the accumulation of mitochondria, which compromises the sarcomeric structure of the cardiomyocytes [113]. Taken together, these findings indicate that Mfn1/Mfn2 double knockout results in mitochondrial fragmentation and stronger cardiotoxicity than deletion of only one of the Mfns.

### 4.3. Mff

Mff-deficient mice exhibit impaired mitochondrial function and increased mitophagy, leading to dilated cardiomyopathy and heart failure by 13 weeks of age. This cascade leads to a decrease in cardiac ATP concentration, which predisposes cardiomyocytes to apoptosis, culminating in fibrotic changes and heart failure. Surprisingly, concomitant deletion of Mfn1 attenuates these pathological changes, preserved cardiac function and extended lifespan [122].

### 4.4. Opa1

Cardiomyocyte-specific heterozygous Opa1-deficient mice do not show cardiac abnormalities under non-stress conditions, but they show enlarged mitochondria. Furthermore, in vitro studies on these cardiomyocytes reveal a reduced sensitivity of the opening of the mitochondrial permeability transition pore (mPTP) to calcium accumulation [123].

**Table 1:** Animal models of mitochondrial morphology factors.

Gene	Model	Phenotype	Mitochondrial Morphology	Reference
*Drp1*	Drp1 deletion homo	Embryonically lethal	Reduced mitochondrial fission	[107]
	Drp1 deletion hetero	Similar to WT		
	Cardiac Drp1 deletion (early postnatal)	Lethal	Enlarged, heterogeneity	[109]
	Drp1 deletion (muscle-specific)	Lethal, Dilated heart		[110]
	Cardiac Drp1 KO (postnatal)	Lethal, Dilated heart	-	[111]
	Cardiac Drp1 KO using tamoxifen (ad 8 weeks)	DCM, Fibrosis	Enlarged	
	Cardiac Drp1 KO using tamoxifen (ad 15 weeks)	Hypertrophy, Fibrosis	Elongated	[108]
	Cardiac Drp1 KO using tamoxifen (ad 8 weeks)	DCM	Enlarged	[112]
	Overexpression of Drp1 (Transgenic mice)	Not deleterious	Fragmented	[113]
*Mfn1/2*	Mfn1/2 KO	Embryonically lethal	-	[124]
	Cardiac Mfn1/2 KO (mid-gestational and postnatal)	Normal at birth Lethal DCM	Spherical, heterogeneity	[114]
	Cardiac Mfn1/2 KO (embryonic)	Cardiac development failure	Fragmented	[125]
	Cardiac Mfn1/2 KO using tamoxifen (early embryonic)	Lethal	Fragmented	[115]
	Cardiac Mfn1/2 KO using tamoxifen (within 8 weeks)	Lethal, DCM		
	Cardiac Mfn1/2 KO (8 weeks–10 weeks)	Impaired myocardial function	Fragmented	[116]
	Marf (*Drosophila*)	DCM	Spherical, Fragmented, heterogeneity	[121]
*Mfn1*	Cardiac Mfn1 deletion	Normal	Spherical, small	[117,118]
	Cardiac Mfn1 deletion	Normal	Large	[126]
*Mfn2*	Cardiac Mfn2 deletion	DCM	Enlarged	[118,119]
	Cardiac Mfn2 deletion	Normal	Heterogeneity	[126]
*Drp1/Mfn1/Mfn2*	Cardiac triple knockout Mfn1/Mfn2/Drp1	HCM	Fragmented	[113]
*Mff*	Mff deletion	DCM	No changes, heterogeneity	[122]
*Opa1*	Opa1 KO	Embryonically lethal	-	[127]
	Heterozygous Opa1 KO	Normal	Enlarged	[123]

Abbreviations: DCM—Dilated cardiomyopathy; HCM—Hypertrophic cardiomyopathy.

## 5. Cardiomyopathy

Several mitochondrial morphology factors have been implicated in the complex relationship between mitochondrial dynamics and cardiomyopathy, heart failure, and reperfusion injury. Here, we focus on five key molecules—Drp1, Mfn1/2, Mff and Opa1.

### 5.1. Drp1

Mitochondrial dynamics between fission and fusion are finely regulated in response to nutrient availability and metabolic demands. C57BL/6 mice develop hyperlipidemia and hyperglycemia when exposed to a high-fat diet. This condition is caused by activation of Drp1 at serine 616, which leads to myocardial insulin resistance, reduces contractile efficiency, and ultimately cardiomyocyte death [128]. The accumulation of lipids results in an increase in mitochondrial ROS and affects the activity of Drp1. This is characterized by a decrease in phosphorylation at serine 637, along with an increase in phosphorylation at serine 616 [129]. In addition, hyperglycemia causes Drp1-mediated mitochondrial fragmentation, resulting in increased ROS production, which adversely affects mitochondrial energy production [130]. Hyperglycemia activates Drp1 phosphorylation at serine 616 and induces mitochondrial fission through Ca2+-mediated ERK1/2 signaling in cardiac myoblast cells [131]. In cardiomyocytes from Zucker diabetic rats, the expression of Opa1 and Mfn2 is decreased, and the phosphorylation of Drp1 at serine 637 is reduced. Conversely, Drp1 phosphorylation at serine 616 is increased, resulting in cardiomyocyte hypertrophy with abnormal mitochondrial dynamics and calcium handling due to activation of the ORAI calcium release-activated calcium modulator 1 (Orai1) calcium channel [132]. In neonatal rat cardiomyocytes, hyperglycemia downregulates Opa1 and Mfn1 but upregulates Drp1 and Mfn2, affecting mitochondrial potential and increasing apoptosis [133]. In human cardiomyocytes, diabetes-associated glycation end products stimulate ERK1/2 and O-linked-N-acetylglucosamine glycosylation [134]. This glycosylation modulates Opa1 expression and Drp1 phosphorylation, which promotes mitochondrial fragmentation in diabetic mouse cardiomyocytes [27,135]. In cardiomyocytes, Drp1-regulated mitochondrial fragmentation is associated with insulin resistance. Drp1 knockdown in H9C2 cells attenuates H_2_O_2_-induced mitochondrial dysfunction [136]. In addition, lipotoxic cardiomyopathy, exacerbated by fatty acid overload, is associated with insulin resistance through ceramide accumulation and upregulation of Drp1 and Mff [137]. In advanced stages of dilated cardiomyopathy, the association with abnormal mitochondrial fragmentation is significant, highlighting the important role of mitochondrial dynamics in the pathological state of the heart [138]. Sepsis reduces myocardial mitochondrial respiration and membrane potential. In addition, the interaction between Drp1 and Fis1 induces ROS production and excessive mitochondrial fragmentation [139].

### 5.2. Mfn1/2

Mitofusins, Mfn1 and Mfn2, are GTPase proteins essential for mediating fusion of the OMM. The prognosis for severe heart failure remains poor. Some heart failure patients do not respond to established multidisciplinary therapy and are classified as “non-responders”. Mfn1 is significantly reduced in non-responders [140]. In patients with diabetic cardiomyopathy, mitochondria in the heart become smaller. This morphological change is associated with decreased hemoglobin A1C levels and associated Mfn1 expression, suggesting that hyperglycemia promotes mitochondrial remodeling [141].

### 5.3. Mff

Mff acts as a receptor for Drp1 on the OMM, facilitating the fission process. Its role is highlighted in lipotoxic cardiomyopathy, where fatty acid overload coupled with ceramide accumulation increases the expression of both Drp1 and Mff, perpetuating mitochondrial dysfunction [137].

### 5.4. Opa1

Opa1 is critical for fusion of the inner mitochondrial membrane. Cardiomyocytes from Zucker diabetic rats show reduced Opa1 expression, which affects hypertrophy and calcium handling via the Orai1 channel [132]. Hyperglycemia decreases Opa1 in neonatal rat cardiomyocytes, reducing mitochondrial potential and increasing apoptosis [133]. Glycation end products generated in human cardiomyocytes increase Opa1 expression and cause mitochondrial fragmentation in diabetic mouse cardiomyocyte models [27,135]. In addition, doxorubicin treatment in FVB/N mice increases Opa1 expression and affects mitochondrial function [142].

## 6. Heart Failure

### 6.1. Drp1 and Mfn2

Cardiac-specific overexpression of miR-122, elevated in heart failure patients, induces mitochondria-dependent cardiomyocyte apoptosis and accelerates heart failure through the activation of Drp1 by inhibiting Hand2 [143]. Drp1 plays a pivotal role in the induction of mitophagy. Drp1-mediated mitochondrial fission is crucial for the onset of mitophagy in the heart [109,144]. The Drp1 C452F mutation in mice exhibits increased Drp1 GTPase activity as well as confers resistance to oligomer degradation, ultimately leading to impaired mitophagy, mitochondrial depolarization, abnormal calcium handling, impaired ATP synthesis, and activation of sterile myocardial inflammation [145]. In mice with heart failure with transverse aortic constriction (TAC), mitochondrial autophagy associated with Drp1 is transiently activated and subsequently downregulated in the mouse heart in response to pressure overload [146]. In a rat heart failure model, cardiomyocytes demonstrate augmented production of reactive oxygen species (ROS) from mitochondria. Notably, the expression levels of Mfn2 and Drp1 are diminished by approximately 50%. Such downregulation leads to an accumulation of mitochondrial Parkin and subsequent induction of mitophagy. This observed regulation of mitochondrial dynamics and mitophagy, mediated through Mfn2 and Drp1, seems to play a pivotal role in cardioprotection, possibly via modulation of ketone body dynamics [147]. HFrEF patient exhibits increased Drp1 levels. Clinically, Mfn2 levels are stable in HFrEF patient samples [148].

### 6.2. Mfn2

Xbp1 expression, a sarcoplasmic reticulum stress-responsive transcription factor, enhances the stress-responsive capacity of the sarcoplasmic reticulum and rescues cardiomyopathy caused by mitofusin/MARF deficiency without ameliorating cardiomyopathy caused by Opa1 deletion [149]. Mitochondrial health in cardiomyocytes is maintained not only by mitochondrial dynamics but also by mitophagy (selective isolation of damaged mitochondria by autophagy). Mitophagy in cardiomyocytes is mediated by Parkin and PTEN-induced kinase 1 (PINK1). PINK1 is stabilized in depolarized mitochondria, allowing its accumulation and facilitating the recruitment of parkin from the cytosol to depolarized mitochondria by Mfn2, an important mediator of mitophagy through the PINK1-Mfn2-perkin signaling pathway [150]. PINK1 phosphorylates Mfn2, and Mfn2 activates parkin [151,152]. Parkin then ubiquitinates Mfn2 [153]. P62 interacts with ubiquitinated substrates marked by Parkin, connecting them to LC3 [112]. Following the elongation of the isolation membrane, mitochondria become encased by this extended membrane, culminating in the formation of an autophagosome. This leads to the subsequent degradation of the ensnared mitochondria [112,144].

### 6.3. Opa1

Cardiomyocyte-specific Yme1L deficient mice exhibit reduced Opa1 levels via activation of Oma1, leading to a shift in cardiac metabolism and, ultimately, heart failure [81]. Ischemic conditions lead to a reduction in Opa1 expression both in vivo and in vitro [154]. In the rat hearts followed for 12 to 18 weeks after myocardial infarction, Mfn2 was reduced, and Fis1 was increased, but Opa1 expression was unchanged [155]. Furthermore, Opa1 mutant heart tissues show increased ROS levels and mitochondrial dysfunction [156]. Ventricular hypertrophy model mice have been shown to have increased expression of Drp1 and reduced expression of Opa1. This indicates that modulating the optimal balance of mitochondrial dynamics can improve mitochondrial function and delay the onset of right ventricular dysfunction [157].

## 7. Ischemia-Reperfusion Injury and Cardioprotection

### 7.1. Drp1

Dynamin-related protein 1 (Drp1) plays a crucial role as an inducer of mitochondrial fragmentation in the detrimental effects of cardiomyocytes after ischemia-reperfusion (I/R) injury. In I/R injury, Drp1 translocates into the OMM and causes mitochondrial fragmentation [158]. Mitochondrial fission inhibitor-1 (mdivi-1), a specific inhibitor of Drp1, administered prior to I/R, inhibits mitochondrial fragmentation, prevents the opening of mitochondrial permeability transition pore, and showed a reduction in cell death and infarct size in a mouse I/R model [93]. Under I/R conditions, both mitochondrial calcium overload and oxidative stress promote mitochondrial fragmentation [159]. After reperfusion, elevated Ca^2^+ strongly regulates Drp1 activity. Notably, miR-499 attenuates the effects of Ca^2^+ overload on Drp1 function [32]. Drp1 has five phosphorylation sites located at serines 585, 616, 637, 656, and 693. Of these, serines 616, 637, and 656 are especially involved in the regulation of Drp1 activation and inactivation under I/R conditions [159] [24,30,160,161,162]. Under conditions of oxidative stress, Drp1 is phosphorylated at Ser 579 by PKCδ, leading to its translocation to the mitochondria [163].

### 7.2. Mfn1 and Mfn2

Mitofusin 1 and 2 (Mfn1/2) are elongation factors of mitochondrial dynamics, and their expression levels are strongly associated with the pathophysiology of I/R injury. Mitochondrial fragmentation induced by I/R also results from decreased expression of Mfn1, Mfn2, or Opa1, leading to impaired respiratory function. Consequently, it promotes I/R-induced cardiomyocyte apoptosis [164,165,166]. I/R stress increases miR-140, inhibits *Mfn1* expression in cardiomyocytes, blocks mitochondrial networks, and exacerbates apoptosis in cardiomyocytes [167]. In vitro experiments with cardiomyocytes, hypoxia upregulates Mfn2 expression [168]. In other in vivo experiments, heart-specific Mfn2 KO Mice exhibit a large reduction in mitochondrial membrane potential and significantly reduced viability under I/R conditions [169]. Additionally, heart-specific Mfn1/Mfn2 double KO Mice exhibit a smaller myocardial infarction size in response to I/R [170]. Overexpression of Mfn1 significantly improves microvascular function under hypoxic conditions, and overexpression of Mfn2 protects cardiomyocytes from I/R injury [171,172].

### 7.3. Opa1

Opa1 has been identified as playing a protective role in cardiomyocytes following I/R injury. A notable downregulation of Opa1 expression is observed in cardiomyocytes subjected to I/R injury in vivo, as well as in hypoxia-treated cardiomyocytes in vitro [154]. Opa1 is reduced in rat heart failure models after MI and in tissue samples of human dilated and ischemic cardiomyopathy [173]. Opa1 heterozygous KO mice display an increased propensity for cardiomyocyte death under I/R conditions, resulting in a larger infarct size post-I/R exposure than their wild-type counterparts [174]. Opa1 overexpression has been linked to the induction of mitophagy, presenting a potential therapeutic avenue for improving cardiomyocyte damage and viability under hypoxic conditions [154].

## 8. Therapeutic Targeting of Mitochondrial Fusion and Fission Proteins

This section summarizes the therapeutic potential for mitochondrial morphological abnormalities. Numerous investigations have explored various agents targeting each mitochondrial regulatory factor (Table 2). Although still in the preliminary stages of experimentation, continued research may further establish mitochondrial morphogenesis as a promising therapeutic target.

### 8.1. Drp1

Melatonin inactivates I/R injury-induced phosphorylation of Drp1 at serine 616. Long-term melatonin treatment attenuates the dilated cardiomyopathy progression and reduces myocardial vulnerability to I/R injury by maintaining mitochondrial quality control. Melatonin membrane receptors are associated with regulating the SIRT6-AMPK-PGC-1α-AKT axis in this effect [175]. Hydralazine reduces myocardial infarct size in cardiac I/R injury by inactivating Drp1 [176]. However, excessive inhibition of mitochondrial fragmentation, as observed with high doses of mdivi-1, which inhibits Drp1 activation, may exacerbate myocardial injury through inhibition of mitophagy and accumulation of abnormal mitochondria [108]. In the area of Drp1 inhibition, P110 and Dynasore emerge as promising therapeutic compound [177,178]. Donepezil inactivates Drp1 phosphorylation at serine 616, indicating its therapeutic potential after I/R injury [179]. Inhibition of Ca2+ entry into mitochondria via Orai1 reduces Drp1-mediated fission during hyperglycemia, providing a therapeutic hint for diabetic cardiomyopathy [132]. Klotho inhibits the phosphorylation of Drp1 at serine 616 and attenuates doxorubicin-related cardiomyopathy [180]. Sevoflurane protects the heart from I/R injury by increasing Drp1 and Parkin and stabilizes ATP levels by ameliorating mitochondrial damage in the rat heart [181]. For arterial calcification, Mdivi-1, melatonin and irisin inhibit arterial calcification by inhibiting mitochondrial fragmentation [182,183,184]. Quercetin, a polyphenol, also improves arterial calcification by inactivating Drp1 phosphorylation at serine 616 [185]. In hypertension, Mdivi-1 is shown to be effective [186,187,188]. As a treatment for pulmonary Hypertension (PAH), dichloroacetic acid, a pyruvate dehydrogenase kinase inhibitor, improves fibrosis and hypertrophy of the right ventricular in rats treated with monocrotaline. This therapeutic effect is mediated through the DNA methyltransferase-1/HIF-1α/PDK/Drp1 pathway [189]. Trimetazidine inhibits hypoxia-induced pulmonary artery smooth muscle cell (PASMC) proliferation by modulating Drp1 and Mfn2 [190]. Liraglutide, a glucagon-like peptide-1 receptor agonist, inhibits PASMC proliferation in PAH via inactivation of the Drp1/NADPH oxidase pathway and by LC3-dependent autophagy [191]. AT1R inhibition also reverses premature aging in VSMC exposed to ox-LDL and in the arteries of ApoE KO mice [192]. In a Huntington’s disease model, the Drp1 inhibitor P110 improves mitochondrial structure in the heart [193].

### 8.2. Mfn1 and Mfn2

SAMβA attenuates cell death through Mfn1 enhancement in ischemic heart failure [194]. In the Wistar rat I/R model, aerobic exercise increases Mfn1 and Mfn2 expression and improves infarct size [195]. Cordycepin also reduces infarct size in diabetic model mice by mitochondrial fusion through the AMPK/Mfn2 pathway [196]. The angiotensin II type I receptor inhibitor and adiponectin mitigate VSMC proliferation via Mfn2-mediated Ras/Raf/ERK signaling [197,198]. Donepezil activates Mfn2 and Opa1, inducing mitophagy, which results in the improvement of ROS production, mitochondrial dysfunction, and cardiac apoptosis under I/R conditions [179]. In hypertension, pomegranate extract mitigates oxidative stress and improves mitochondrial function, coinciding with reduced Mfn2 [199]. Resveratrol increases Mfn1 and Mfn2 expression and reduces oxidative stress damage in human umbilical vein endothelial cells [200]. Fish oil supplementation boosts Mfn2 and Opa1, preventing endothelial dysfunction in high-fat-fed ApoE KO mice [201]. Ferulic acid may restore Mfn1 and Mfn2 levels and attenuate oxidative stress in high-fat-fed ApoE KO mice [202]. In spontaneously hypertensive rat hearts, Drp1 is notably upregulated while Opa1 and Mfn2 decrease. Calhex231, a calcium sensing receptor inhibitor, counters these alterations in mitochondrial dynamics, subsequently reducing apoptosis in hypertensive hearts [203].

### 8.3. Opa1

Irisin administration induces Opa1-mediated mitophagy and protects myocardial cells from post-myocardial infarction damage [154]. Remote ischemic preconditioning enhances Opa1 expression, decreasing myocardial infarct size [166]. Melatonin activates the AMPK/Opa1 axis, promoting mitochondrial fusion and mitophagy, which ameliorates cardiomyocyte death and mitochondrial dysfunction after I/R injury [204]. Paeonol activates Opa1-mediated fusion through the CK2α-STAT3 pathway during diabetes, both in vitro and in vivo [205]. Sevoflurane postconditioning reduces Opa1 expression, protects against I/R injury, stabilizes ATP levels, and rescues mitochondrial damage in the rat heart [181]. Nicorandil suppresses fission and boosts fusion by downregulating Drp1 and upregulating Opa1 and Mfn1 in ischemic cardiomyopathy rats [206]. Coenzyme Q10 promotes mitochondrial function and energy metabolism by activating the AMPK/YAP/Opa1 pathway, attenuating atherosclerosis [207].

**Table 2:** Targeting mitochondrial morphology factors for cardiac disease.

Target Up (+) or Down (−)	Method	Model		Result	Reference
Drp1 (−)	Melatonin	I/R	Diabetic rat with I/R injury	Decrease in MI size and apoptosis	[175]
	Hydralazine		C57BL/6N with I/R injury	MI size reduction	[176]
	P110		Wistar rat, Ex vivo	Improvement of mitochondrial morphology and mitochondrial respiratory function.	[177]
	Dynasore		C6/Black, Ex vivo	Increase in cardiomyocyte viability	[178]
	BTP2	Diabetic CM	Zucker diabetic fat	Improvement of cardiomyocyte hypertrophy	[132]
	Klotho	Dox-CM	C57BL/6 treated with doxorubicin	Inhibition of apoptosis	[180]
	Sevoflurane postconditioning	Ischemic HF	Sprague-Dawley rat with I/R injury	Improvement of ATP production with mitophagy	[181]
	Mdivi-1	ATS	Human VSMC	Suppression of VSMC calcification	[182]
	Melatonin		Rat VSMC	Suppression of arterial calcification	[183]
	Irisin		High-phosphorus-diet C57BL/6	Suppression of arterial calcification	[184]
	Quercetin		Adenine-rich diet rat	Suppression of arterial calcification	[185]
	Mdivi-1	HTN	C57BL/6 treated with AngII	Inhibition of AngII-mediated phenotypic switch	[186]
	Mdivi-1		High-salt-fed rat	Reduction of cardiac hypertrophy and fibrosis	[187]
	Mdivi-1		Rat VSMC	Suppression of arterial calcification	[188]
	Dichlorpacetate	PH	Monocrotaline-treated rat	Inhibition of right ventricular fibrosis and hypertrophy	[189]
	Liraglutide		Rat PASMC	Inhibition of cell proliferation	[191]
	ARB	Aging	Human VSMC and ApoE KO mice	Reduction of hyperlipidemic aging	[192]
	P110		Huntington Disease (HD) model mice	Reduction of pathological mitochondrial fission	[193]
Mfn1 (+)	SAMβA	Ischemic HF	Rat treated with AngII	Inhibition of apoptosis	[194]
Mfn2 (+)	Cordycepin	I/R	Diabetic mice with I/R injury	MI size reduction	[196]
	ARB	ATS	Rat VSMC	Inhibition of cell proliferation	[197]
	Adiponectin	ATS	Human VSMC	Inhibition of cell proliferation	[198]
Mfn2 (−)	Pomegranate	HTN	SHR	Reduction of oxidative stress	[199]
Mfn1 and Mfn2 (+)	Aerobic exercise	I/R	Wistar rat with I/R injury	MI size reduction	[195]
	Ferulic acid	ATS	High-fat-fed ApoE KO mice and Human mononuclear cell	Reduction of oxidative stress	[202]
Mfn1 and Opa1 (+)	Fish oil	ATS	High-fat-fed ApoE KO mice	Improvement of endothelial dysfunction	[201]
Mfn1/2 and Opa1 (+)	Resveratrol	ATS	HUVEC treated with palmitic acid	Improvement of cell viability and reduction of oxidative stress	[200]
Drp1 (−), Mfn2 (+)	Trimetazidine	PH	Human PASMC	Inhibition of hypoxia-induced cell proliferation	[190]
Drp1 (−), Mfn2 and Opa1 (+)	Donepezil	I/R	Wistar rat with I/R injury	Amelioration of apoptosis and mitochondrial dysfunction	[179]
	Calhex231	HTN	SHR	Inhibition of apoptosis	[203]
Drp1 (−), Mfn1 and Opa1 (+)	Nicorandil	Ischemic HF	Rat with I/R injury	Increased mitochondrial ATP-sensitive potassium channel opening	[206]
Drp1 (−), Opa1 (+)	Sevoflurane postconditioning	Ischemic HF	Sprague-Dawley rat with I/R injury	Induction of mitophagy and improvement of myocardial ATP production	[181]
Opa1 (+)	RIPC	I/R	Wistar rat with I/R injury	MI size reduction	[166]
	Irisin		Hypoxia-treated cardiomyocyte	Inhibition of apoptosis	[154]
	Melatonin		C57BL/6 with I/R injury	Amelioration of apoptosis and mitochondrial dysfunction	[204]
	Paeonol	Diabetic CM	Sprague-Dawley rat cardiomyocytes under high glucose condition	Improvement of cardiomyocyte hypertrophy and interstitial fibrosis	[205]
	Coenzyme Q10	ATS	High-fat-fed ApoE KO mice	Inhibition of oxidative stress and promotion of energy metabolism	[207]

Abbreviations: +—upregulation; −—downregulation (inhibition); ARB—Angiotensin II type I receptor inhibitor; MI—myocardial infarct; HUVEC—human umbilical vein endothelial cell; RIPC—remote ischemic preconditioning; CM—cardiomyopathy; Dox-CM—doxorubicin-associated cardiomyopathy; ATS—Atherosclerosis; HTN—hypertension; HF—Heart failure; SHR—spontaneously hypertensive rat. The table was modified from Yoshihiro et al. [208].

## 9. Conclusions

Mitochondrial dynamics, including fission and fusion processes, play an essential role in heart health and disease. Recent research has highlighted the potential of targeting these dynamics to treat various cardiac diseases, including acute myocardial infarction, hypertrophy, PAH, ischemic diseases and heart failure. However, it is important to recognize the multifaceted role of mitochondrial morphology factors, particularly fusion proteins such as Mfn2 and Opa1. Acute and chronic modulation of these proteins may have therapeutic effects or unintended adverse consequences. Although most of the current knowledge is derived from transgenic animal models, early pharmacological interventions have yielded promising results. It is essential to extend this research to human samples and to improve the diagnostic and therapeutic toolkit by integrating biopsy analysis and indirect markers of mitochondrial dynamics. This would help to refine patient risk assessment and tailor treatment strategies for cardiovascular disease.

Furthermore, there is a perception that mitochondrial fusion is beneficial and fission is detrimental, but this binary view may be too simplistic. Fission plays a fundamental role in separating damaged mitochondria for removal by mitophagy, and excessive inhibition of this process may exacerbate myocardial injury. Thus, striking the right balance in the regulation of mitochondrial dynamics is critical for potential clinical applications.

## Data Availability

Not applicable.
